# Personality Traits in Fibromyalgia: Aggravators and Attenuators of Clinical Symptoms and Medication Use

**DOI:** 10.1155/bn/9961041

**Published:** 2025-07-15

**Authors:** Dolores Santiago, Casandra I. Montoro, Dmitry M. Davydov, Gustavo A. Reyes del Paso

**Affiliations:** Department of Psychology, University of Jaén, Jaén, Spain

**Keywords:** fibromyalgia syndrome, medication use, personality, severity of clinical symptoms

## Abstract

**Introduction:** Patients with fibromyalgia syndrome (FMS) exhibit higher levels of neuroticism and psychoticism and lower levels of extraversion, which may influence medication use.

**Objective:** The objective of this study was to analyze associations between personality traits (from Eysenck's model) and medication use in patients with FMS and to explore factors mediating/moderating these relations.

**Method:** Data on personality, medication use, and clinical severity were collected from 94 FMS patients and 56 individuals from a nonclinical population.

**Results:** Patients had higher neuroticism and psychoticism compared to participants from the nonclinical population, with no significant differences observed in extraversion. Neuroticism was positively associated with the use of antidepressants and anxiolytics, as well as with higher levels of anxiety and depression. In contrast, extraversion was negatively associated with the use of antidepressants, anxiolytics, and opioids and with lower anxiety, depression, and the emotional and cognitive dimensions of pain and a reduced prevalence of comorbid emotional disorders. Psychoticism was positively associated with the sensorial, emotional, and cognitive dimensions of pain. Depression and anxiety levels mediated the relationships between neuroticism and extraversion and the use of antidepressants and anxiolytics. Additionally, comorbid depressive disorders moderated the association between neuroticism and antidepressant use.

**Conclusion:** Higher neuroticism and lower extraversion primarily increase the likelihood of using mood-regulating medications, but not analgesic drugs. Implementing coping strategies aimed at reducing neuroticism and enhancing extraversion may help to reduce medication use in patients with FMS.

## 1. Introduction

Fibromyalgia syndrome (FMS) is characterized by persistent and widespread musculoskeletal pain, along with the presence of hyperalgesia and allodynia [1]. FMS is accompanied by stiffness, fatigue, sleep problems, cognitive disturbances, migraines, irritable bowel syndrome, paresthesia, etc. [2–4], leading to a considerable reduction in quality of life [5, 6]. The etiology of FMS remains unclear, although evidence points to an anomaly in central pain processing and descending pain inhibitory mechanisms, that is, to a disorder of central sensitization that amplifies pain [7–10].

FMS is usually accompanied by depression and anxiety [11–18]. Major depressive disorder and anxiety disorders are very common in FMS, with a prevalence in the range of 50%–70% [19]. Personality shapes how individuals perceive their environment and respond to life events, including the experience of pain, thus being a relevant factor in the development of emotional disorders among patients with FMS [11]. Personality is defined as the combination of stable characteristics related to affect, cognition, and behavior [16], reflecting the consistent patterns of thinking and acting across various stimuli and situations over time [6]. Individual differences in the appraisal of life experiences play a significant role in pain management [17]. Personality influences the adoption of beliefs and coping strategies in response to pain, shaping both the experience and evaluation of pain [18, 20], as well as patterns of medication use [21]. FMS symptoms are partly influenced by dysfunctional behavior patterns and negative affect, both of which are core components of personality [3]. Thus, personality can influence FMS symptoms, potentially intensifying or alleviating them and thereby modulating the overall pain experience [11].

H.J. Eysenck's personality model comprises three major traits: neuroticism, extraversion, and psychoticism [22]. Neuroticism and extraversion have been the most extensively studied personality traits in relation to FMS [3, 14, 16, 23], with evidence suggesting that they may modulate FMS symptomatology [14, 24]. Neuroticism is characterized by a tendency to experience negative emotions such as fear, anxiety, sadness, anger, and guilt [25] and is associated with passive and maladaptive coping beliefs and strategies [8, 21]. This trait contributes to heightened arousal during pain anticipation and avoidance behavior during pain stimulation [24], as well as catastrophizing and dysfunctional interpretations of pain [26, 27]. Patients with FMS have been found to exhibit higher levels of neuroticism or, more broadly, increased negative affectivity [11, 14, 28]. Extensive evidence indicates that neuroticism influences pain perception [29, 30], promoting the use of maladaptive coping strategies that, in turn, exacerbate FMS symptoms [5, 21]. Neuroticism has also been linked to comorbid depression and anxiety in FMS [3]. Numerous studies have reported significantly higher levels of neuroticism in women compared to men [29], which aligns with the higher prevalence of FMS among women [1, 4].

With respect to extraversion, individuals high in this trait tend to engage in more positive evaluations, focus on external stimuli, and are oriented toward reward-seeking behavior. These characteristics are considered protective factors that may reduce pain sensitivity [22, 31]. Research has shown that individuals from nonclinical populations with high levels of extraversion exhibit reduced physiological reactivity to painful stimuli [32]. Although lower levels of extraversion have been observed in FMS patients [16], this finding has not been consistently replicated across all studies (e.g., [3] and [25]). Regardless, patients with FMS higher in extraversion tend to report lower clinical pain intensity and greater pain tolerance [33]. Higher extraversion is also associated with reduced anxiety and fewer functional impairments in the daily life. Consequently, extraversion has been identified as a potential protective factor that may attenuate the severity of FMS symptoms [3, 14, 19, 34].

Finally, higher levels of psychoticism characterized in this framework by features such as impulsivity, aggressiveness, egocentrism, irresponsibility, and emotional detachment have been observed in patients with FMS compared to nonclinical populations [14, 21, 23, 35, 36]. Moreover, while greater psychoticism has been associated with catastrophizing-related coping strategies in nonclinical populations, this association has not been found in patients with FMS [14].

Pharmacological interventions for FMS primarily target symptoms through the use of various analgesics, including nonsteroidal anti-inflammatory drugs (NSAIDs) and, in some cases, opioids [37]. Antidepressants and anxiolytics are also commonly prescribed as part of the treatment regime [38]. Antidepressants are used not only to alleviate depressive symptoms commonly observed in FMS, but also for their analgesic properties, which are attributed to their modulation of neurobiological pathways shared by both pain and depression particularly the serotonergic and noradrenergic systems [39]. In general, high levels of medication use are observed in FMS patients [40]. The relationship between personality and medication use in FMS has been previously explored, primarily in the context of personality's moderating role in the association between stress-coping mechanisms and medication use [21]. Personality can influence the cognitive resources available for selecting effective stress-coping mechanisms, which may, in turn, reduce the reliance on pharmacological treatment. Research on substance use and addiction disorders indicates that affected individuals often exhibit higher levels of neuroticism and lower levels of extraversion [41–45]. Additionally, neuroticism (positively) and extraversion (negatively), as measured by the Big Five Inventory, were found to predict the use of opioids and mood-regulating medications for pain management over a 10-year follow-up in individuals over the age of 50 with persistent pain [46].

The aim of this study was to assess the associations between the personality traits defined in H.J. Eysenck's personality model and the use of medications including antidepressants, anxiolytics, nonopioid analgesics, and opioids in patients with FMS. The severity of clinical symptoms (i.e., the intensity of pain, insomnia, fatigue, depression, and anxiety) as well as comorbid depression and anxiety disorders was assessed for their potential mediating and moderating effects on these associations. The following hypotheses were tested: (1) Neuroticism would be positively associated with medication use; (2) extraversion would be inversely related to medication use; (3) neuroticism (positively) and extraversion (negatively) would be associated with the severity of FMS symptoms and the prevalence of comorbid emotional disorders; and (4) the severity of FMS symptoms and comorbid emotional disorders would mediate and moderate, respectively, the relationship between the personality traits and medication use.

## 2. Method

### 2.1. Participants

Ninety-four patients (three males) diagnosed with FMS by rheumatologists according to the 1990 criteria of the American College of Rheumatology (ACR; [47]), and 56 individuals (two males) from a nonclinical population, recruited to serve as a comparison control group, participated in the study. The predominantly female composition of the sample reflects the significantly higher prevalence of FMS in women compared to men [47, 48]. Exclusion criteria for the participants in both groups included the presence of cardiovascular, inflammatory, metabolic, oncological, or neurological conditions, as well as severe psychiatric disorders such as psychosis or substance use disorders. For the nonclinical participants, an additional exclusion criterion was the presence of any chronic pain–related condition. Age, years of education, and body mass index (BMI) were comparable between the two groups, as presented in Table 1.

### 2.2. Procedure

Patients with FMS were recruited through the Fibromyalgia Association of Jaén (Spain), a nonprofit association created by patients themselves. The association invited its members to participate in the study. Those who expressed interest were contacted by telephone to explain the study's purpose and schedule an appointment. Nonclinical control participants were recruited through acquaintances of the Fibromyalgia Association, neighborhood associations, social media platforms, and campus announcements. The evaluation began with semistructured interview, to assess the inclusion and exclusion criteria, gather sociodemographic data, and record current medication use (classified into antidepressants, anxiolytics, and opioids). Then, the Structured Clinical Interview for Axis I Disorders of the Diagnostic and Statistical Manual for Mental Disorders (SCID; [49]) was administered to identify comorbid mental disorders, especially anxiety and depressive ones. Subsequently, the following validated instruments (Spanish versions) were administered in an interview-based format: (1) Revised-Abbreviated Eysenck Personality Questionnaire (EPQR-A) assesses neuroticism, extraversion, psychoticism, and social desirability (sincerity) through 24 dichotomous (yes/no) items with subscale scores ranging from 0 to 6. Internal consistency (Cronbach's *α*): neuroticism = 0.74, extraversion = 0.78, psychoticism = 0.63, and sincerity = 0.54. Originally developed by Francis et al. [50] and adapted to Spanish by Sandín et al. (2002) [51], the EPQR-A is well suited for clinical settings due to its brevity and demonstrated validity in comparison with the full EPQ. (2) McGill Pain Questionnaire (MPQ; [52]) measures clinical pain severity using 84 items across four subscales: sensory (range: 0–41), affective/emotional (range: 0–9), cognitive (range: 0–4), and miscellaneous (range: 0–12). Cronbach's *α* for the total score = 0.74. (3) Fatigue Severity Scale (FSS; [53]) assesses fatigue through nine items, with total scores ranging from 9 to 63. Cronbach's *α* = 0.88. (4) Oviedo Quality of Sleep Questionnaire (OQS; [54]) evaluates insomnia severity using nine items, with scores ranging from 9 to 45. Cronbach's *α* = 0.77. (5) State-Trait Anxiety Inventory (STAI; [55]) measures both state (current) and trait (habitual) anxiety using 20 items for each subscale (score range: 0–60). Cronbach's *α* = 0.93 (state anxiety) and 0.87 (trait anxiety). (6) Beck Depression Inventory (BDI; [56]) assesses depressive symptom severity through 21 items, with total scores ranging from 0 to 63. Cronbach's *α* = 0.95. All participants provided written informed consent, and the study protocol was approved by the Ethics Committee of the University of Jaén. Participation in the study was voluntary, and no financial compensation was offered.

### 2.3. Statistical Analysis

The Kolmogorov–Smirnov and Levene tests indicated no significant deviations from normality or homogeneity of variance for the measured variables (*p* > 0.05). Chi-square tests were conducted to examine group differences between FMS patients and nonclinical control participants on dichotomous variables (i.e., medication use and presence of comorbid depression or anxiety disorders). For continuous variables (i.e., personality traits and FMS symptoms), group comparisons were conducted using ANOVA models. Effect sizes for these analyses were reported using partial eta squared (*η*_*p*_^2^). Associations between personality traits and other variables were examined using point-biserial correlations for dichotomous variables and Pearson bivariate correlations for continuous variables. Binary logistic regression analyses were conducted to evaluate the predictive capacity of personality traits and comorbid depression/anxiety disorders on medication use, as well as to assess the predictive role of personality in the presence of comorbid depression or anxiety disorders. Nagelkerke's *R*^2^ was used to indicate effect size in these analyses, reflecting the proportion of variance in medication use explained by personality traits. To assess the predictive value of personality for continuous clinical variables, multiple linear regression models were computed using a forward stepwise procedure. Effect sizes for these models were reported using adjusted *R*-squared values. To examine the potential moderating role of comorbid depressive and anxiety disorders in the relationship between personality traits and medication use, linear regression analyses were conducted including interaction terms (personality trait × presence of a depressive or anxiety disorder). Finally, mediation analyses were performed to evaluate whether symptom severity mediated the relationship between personality and medication use. These analyses were conducted using the PROCESS macro for SPSS [57], applying a simple mediation model with 5000 bootstrapping samples and 95% confidence intervals.

All results reported below remained consistent after excluding male participants from the analyses (*n* = 91 for the FMS group and *n* = 54 for the nonclinical control group).

## 3. Results

Patients with FMS reported significantly higher levels of anxiety, depression, fatigue, and insomnia, as well as greater pain severity, compared to nonclinical participants. Additionally, they presented with higher rates of comorbid depressive and anxiety disorders and a greater use of antidepressants, anxiolytics, nonopioid analgesics, and opioids (Table 1).

### 3.1. Group Differences in Personality

Patients with FMS scored significantly higher on neuroticism and psychoticism compared to nonclinical participants, with a particularly large effect size observed for neuroticism. Although FMS patients showed lower levels of extraversion than the nonclinical group, this difference did not reach significance (Table 1). In the FMS sample, neuroticism and extraversion were inversely correlated (*r* = −0.24, *p* = 0.020), whereas no such relationship was observed in the nonclinical group (*r* = −0.06). No other significant correlations emerged among the personality traits.

### 3.2. Associations Between Personality, Medication Use, and Clinical Variables

**Figure 1:**
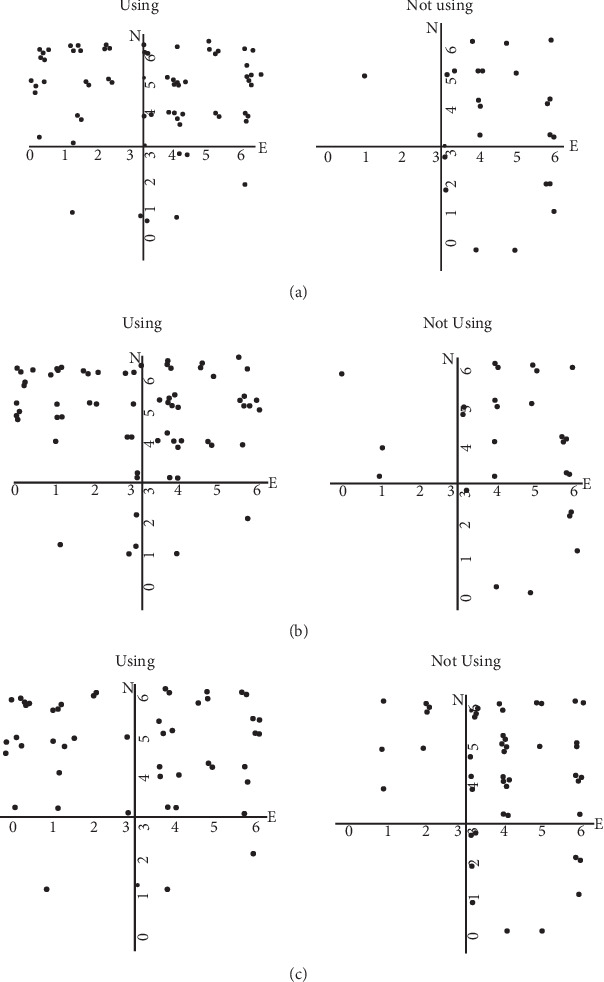
Distribution of patients with FMS using (left) and not using (right) (a) antidepressants, (b) anxiolytics, and (c) opioids as a function of neuroticism (*N*) and extraversion (*E*) scores.

Table 2 presents the point-biserial correlations between personality traits, medication use, and comorbid anxiety and depression disorders in patients with FMS. Higher neuroticism levels were significantly associated with increased use of antidepressants and anxiolytics. In contrast, greater extraversion was linked to lower usage of antidepressants, anxiolytics, and opioids, as well as a lower prevalence of comorbid anxiety disorders.  Figure 1 illustrates medication use across varying levels of personality traits in patients with FMS.

Neuroticism scores were positively correlated with state and trait anxiety, depression, and fatigue. In contrast, extraversion scores showed negative associations with state and trait anxiety, depression, and the emotional and evaluative–cognitive dimensions of pain. Psychoticism was positively correlated with all pain dimensions, including sensory, emotional, evaluative–cognitive, and miscellaneous components (Table 2).

### 3.3. Binary Logistic Regressions Between Personality, Medication Use, and Comorbid Disorders

The use of antidepressants was significantly predicted by the presence of depressive disorders (*B* = 2.11, SE = 0.59, Wald = 12.79, and *p* < 0.001), higher levels of neuroticism (*B* = 0.45, SE = 0.19, Wald = 5.52, and *p* = 0.019), and lower levels of extraversion (*B* = −0.39, SE = 0.19, Wald = 4.28, and *p* = 0.039), with the full model explaining 42% of the variance (*R*^2^ = 0.42). The model showed greater sensitivity (correctly identifying patients using antidepressants) than specificity (correctly identifying those not using them) (Table 3(a)). Furthermore, a significant interaction was found between neuroticism and comorbid depressive disorders in predicting antidepressant use (neuroticism × depressive disorders interaction term: *B* = −0.13, SE = 0.05, *t* = −2.64, and *p* = 0.01), indicating a moderating effect. Specifically, neuroticism significantly predicted antidepressant use in patients without comorbid depression (*B* = 0.15, SE = 0.04, *t* = 3.34, *p* = 0.002, and *r* = 0.52) but not in those with comorbid depressive disorders (*B* = 0.02, SE = 0.03, *t* = 0.56, *p* = 0.57, and *r* = 0.07).

The use of anxiolytics was predicted by the presence of anxiety disorders (*B* = 1.31, SE = 0.51, Wald = 6.46, and *p* = 0.011] and lower levels of extraversion (*B* = −0.33, SE = 0.16, Wald = 4.64, and *p* = 0.031), with the full model accounting for 27% of the variance (*R*^2^ = 0.27). The model demonstrated high sensitivity (accurate identification of anxiolytic users) but low specificity (less accurate identification of nonusers) (Table 3(b)). In contrast, opioid use was predicted solely by the presence of comorbid depressive disorders (*B* = 1.13, SE = 0.48, Wald = 5.46, and *p* = 0.019), with a smaller effect size (*R*^2^ = 0.14). The discrimination rate was again higher for patients taking opiates (i.e., sensitivity) than for those not taking them (i.e., specificity) (Table 3(c)). Logistic regression analysis revealed that neither personality traits nor the presence of mental comorbidities predicted the use of nonopioid analgesics. Finally, extraversion significantly predicted the presence of anxiety disorders, with lower levels of extraversion associated with a higher likelihood of having an anxiety disorder (*B* = −0.23, SE = 0.12, Wald = 0.40, and *p* = 0.045; *R*^2^ = 0.06). The overall correct classification rate for comorbid anxiety disorders was 61.7%, with sensitivity exceeding specificity (Table 3(d)).

### 3.4. Linear Regression Between Personality and Clinical Variables

Neuroticism emerged as the primary positive predictor of state and trait anxiety, depression, and fatigue. Extraversion, showing an inverse association, was the main predictor of the cognitive–evaluative component of pain and also contributed to prediction of trait anxiety, depression, and the emotional component of pain in secondary models. Psychoticism was the strongest predictor of the sensory, emotional, and miscellaneous components of pain. In secondary models, psychoticism, together with extraversion, predicted cognitive–evaluative pain, while extraversion, in combination with psychoticism, contributed to the prediction of emotional pain (Table 4).

### 3.5. Impact of Clinical Symptoms as Mediators of the Relationships Between Personality Traits and Medication Use in Patients With FMS

**Figure 2:**
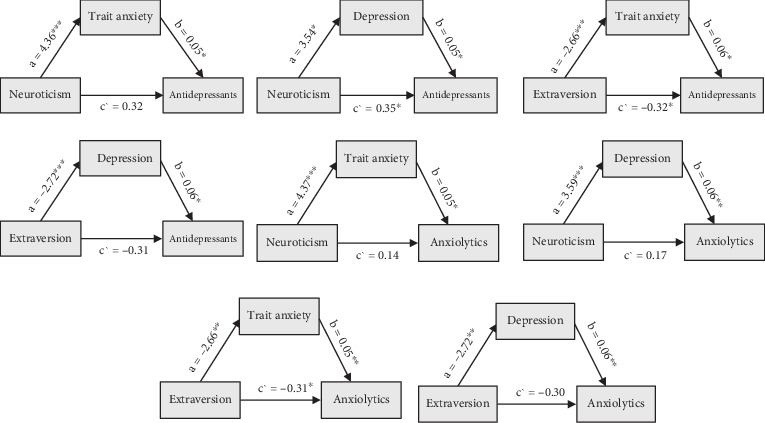
Path diagrams illustrating the mediation effects of trait anxiety and depression on the relationships between neuroticism and extraversion and the use of medications. Note: *a* = path from the predictor to the mediator. *b* = path from the mediator to the dependent variable. *c*′ = direct effect of the predictor on the independent variable accounting for the mediator. ⁣^∗^*p* < 0.05; ⁣^∗∗^*p* < 0.01.

Table 5 summarizes the mediating factors underlying the associations between personality traits and medication use. All mediation effects met Baron and Kenny's criteria: (1) a significant relationship between the independent and dependent variables, (2) a significant relationship between the independent variable and the mediator, and (3) a significant relationship between the mediator and the dependent variable when both the independent variable and the mediator were included in the model. Additionally, the strength of the relationship between the predictor and outcome was reduced when the mediator was controlled for, supporting mediation. Trait anxiety and depression scores mediated the relationships between neuroticism and extraversion, as predictor factors, and the use of anxiolytics and antidepressants as outcomes.  Figure 2 illustrates these mediation pathways.

## 4. Discussion

The aim of this study was to clarify the relationship between personality traits and medication use in patients with FMS, a topic previously explored only indirectly through its association with stress-coping mechanisms [21]. In line with earlier research [11, 12, 14, 28, 35], patients with FMS exhibited higher levels of neuroticism and psychoticism compared to the nonclinical population. However, consistent with previous findings [3, 14, 25], no significant differences were observed in extraversion. Among FMS patients, higher neuroticism scores were associated with greater use of antidepressants and anxiolytics, as well as increased levels of fatigue, anxiety, and depression. In contrast, higher extraversion was linked to lower usage rates of antidepressants, anxiolytics, and opioids; lower levels of anxiety and depression; reduced intensity in emotional and evaluative components of pain; and fewer comorbid anxiety disorders. Additionally, elevated psychoticism scores were associated with more severe clinical pain across sensory, emotional, evaluative–cognitive, and miscellaneous dimensions. Finally, consistent with previous studies [4, 11, 58], patients with FMS showed a higher likelihood of comorbid depressive and anxiety disorders compared to the nonclinical participants.

As anticipated, higher levels of neuroticism and lower levels of extraversion were associated with an increased likelihood of antidepressant and anxiolytic use in patients with FMS. Binary logistic regression analysis indicated that the presence of depressive disorders, along with higher neuroticism and lower extraversion, significantly predicted antidepressant use. Notably, the presence of depressive disorders moderated the relationship between neuroticism and antidepressant use: Higher neuroticism significantly predicted antidepressant use only among FMS patients without comorbid depressive disorders, but not among those with such comorbidity. The presence of anxiety disorders and extraversion (inversely) predicted the use of anxiolytics. Similarly, high extraversion was also associated with reduced opioid use and a lower likelihood of suffering comorbid anxiety disorders. However, in the binary logistic regression model, only the presence of comorbid depression emerged as a significant predictor of opioid use. It is also worth highlighting that personality traits demonstrated greater sensitivity than specificity in identifying patients using antidepressants and anxiolytics; that is, personality factors were more effective at detecting users than nonusers.

In interpreting these results, it is important to highlight that neuroticism and extraversion showed a weak inverse correlation within the patient sample; that is, patients with high neuroticism scores tended to have lower extraversion scores, and vice versa. Supporting our results, a study using the Big Five Inventory found that the combination of high neuroticism and low extraversion predicted more persistent pain and prolonged opioid use during follow-up in nonclinical individuals, both with and without pain at the time of assessment [46]. By contrast, another study did not observe any associations between personality traits, as measured by the Eysenck Personality Questionnaire, and medication use among patients with chronic back pain [38]. This discrepancy may be explained by differences in the type of pain condition studied or by the higher age range of participants (up to 87 years) [38], as medication use tends to be significantly higher in older individuals, irrespective of personality traits [59]. Notably, in our study, personality traits were not associated with the use of nonopioid analgesics, but they were linked to medications targeting emotional or affective symptoms, such as antidepressants and anxiolytics. This finding aligns with existing literature suggesting that personality, particularly neuroticism and extraversion, primarily influences affective states [59]. Likewise, prior studies have reported that individuals with depression are more likely to use opioids over extended periods compared to those without depression [60], which supports our binary regression findings regarding predictors of opioid use.

The opposite directions of the associations between neuroticism and extroversion with symptom severity may help to explain their inverse relationships with the use of antidepressants and anxiolytics, as well as the negative relationship between extraversion and opioid use. However, no significant associations were found between personality traits and the presence of comorbid depressive disorders. This finding contrasts with previous literature, which has identified links between low extraversion and high neuroticism and greater difficulties in emotion regulation [61], difficulties that are, in turn, associated with symptoms of anxiety and depression [62, 63]. The negative impact of neuroticism on FMS symptomatology and mood-regulating medication use may be attributed to its characteristic features, including a persistent negative emotional state, excessive worry and rumination on past and future events, heightened anticipatory anxiety, harm avoidance, self-focused attention and criticism, and increased vigilance to body symptoms and negative interpretations of such sensations [64]. In contrast, the associations between extraversion and lower levels of anxiety and depression, and the emotional and evaluative–cognitive dimensions of pain, support the proposed protective role of extraversion in health outcomes [14], particularly with regard to pain sensitivity [22, 31]. Interestingly, the association between extraversion and comorbid emotional disorders in this study was limited to anxiety disorders, with no significant relation to depressive disorders. This finding is somewhat unexpected, given previous research suggesting that low extraversion is associated with an increased risk for both depressive and anxiety disorders in in individuals with FMS [3]. Nevertheless, the observed relationship between a higher extraversion and a lower prevalence of comorbid anxiety disorders may reflect the role of extraversion in facilitating more efficient emotional regulation [65, 66] and reduced cognitive–emotional reactivity to mild mood fluctuations, namely, a lower tendency to develop maladaptive thought patterns [67].

In this study, lower levels of neuroticism and higher levels of extraversion were associated with reduced use of anxiolytics and antidepressants. These associations may be partly explained by the opposing influence of these personality traits on symptoms of anxiety and depression. The high prevalence of anxiety and depression among individuals with FMS may contribute to the elevated use of anxiolytics and antidepressants. In our sample of 94 patients, 61 were diagnosed with depression and 54 with anxiety disorders. Notably, in the absence of depressive disorders, higher levels of neuroticism were associated with increased antidepressant use. However, when depressive disorders were present, the affective condition itself likely determining doctor-driven prescription appeared to account for the increased use of antidepressants, thereby overshadowing the influence of personality traits such as neuroticism.

Although neuroticism was associated with anxiety, depression, and fatigue in the present study, it was not found to be related to pain severity among FMS patients. Similar null findings have been reported in other studies [68]. However, contrary to our results, several investigations have identified associations between neuroticism and pain in both nonclinical populations and individuals with chronic pain conditions [6, 26, 29, 69, 70]. These discrepancies may stem from differences in patient characteristics, particularly the heterogeneity within FMS populations regarding the mechanisms underlying pain chronicity, or from variations in the assessment tools employed. Notably, previous studies used different instruments to measure both personality traits, which may account for inconsistencies in findings [21].

Inverse associations between extraversion and current pain intensity in patients with FMS have been previously reported [14, 34]. In the present study, such associations were confined to the emotional and evaluative–cognitive dimensions of chronic pain, supporting the notion that extraversion may not directly influence pain mechanisms but rather affects how pain is perceived and interpreted [71]. Individuals high in extraversion may evaluate pain as less threatening and are more likely to adopt effective adaptive coping strategies, potentially including a “fake good” response style, characterized by the minimization or denial of problems [21, 72–74]. This interpretation is consistent with core characteristics of extraversion, including positive affectivity, optimism, stronger interpersonal relationships (i.e., greater social support), elevated activity levels, greater outward focus, heightened reward sensitivity, more favorable cognitive appraisals, and lower reactivity to painful stimuli [22, 75].

This study is the first to identify a positive association between psychoticism and clinical pain severity in patients with FMS. This relationship may be explained by features of the psychoticism trait, such as a tough-minded disposition, impulsivity, aggressiveness, egocentrism, and emotional detachment [22]. Psychoticism has also been linked, particularly in nonclinical populations, to maladaptive pain–related coping strategies, such as catastrophizing [14], which is known to amplify somatic complaints and pain perception [29]. Consequently, individuals high in psychoticism may display a maladaptive affect-regulation profile, potentially characterized by a “fake bad” response style marked by the exaggeration or overreporting of symptoms, help-seeking behaviors, and heightened sensitivity to distress which may contribute to inflated perceptions or reports of pain severity [76, 77].

Regarding the mechanisms linking personality traits with medication use, personality may influence both the expression of symptoms and the nature of patient physician interactions, thereby affecting the type of medications prescribed. Our findings suggest that higher levels of neuroticism and lower levels of extroversion are associated with more pronounced affective symptoms, which may prompt physicians to prescribe mood-regulating medications such as antidepressants or anxiolytics. Additionally, individuals with certain personality profiles may perceive specific medications as ineffective and request alternative treatments. As the current study did not assess medication effectiveness, this latter possibility warrants investigation in future research.

This study has some limitations. First, it relied on the EPQR-A. Although validated for clinical use, some subscales—such as psychoticism and sincerity—demonstrate relatively modest reliability indices. Additionally, the use of this abbreviated version may limit the depth and comprehensiveness of the personality assessment. Nonetheless, despite comprising only six items per dimension, the EPQR-A has demonstrated strong overall psychometric reliability and validity [52, 53]. Another limitation is the omission of coping strategy assessments, such as catastrophizing, avoidance, and cognitive distraction, which are known to influence both pain perception and medication use [21]. Future research should incorporate these factors to better understand their impact on medication use in chronic pain populations. Moreover, our findings may not be directly generalizable beyond patients with FMS. The limited number of male participants also constitutes a limitation; however, this reflects the well-documented epidemiology of FMS, which predominantly affects women [47, 48]. Importantly, the analyses conducted exclusively with female participants yielded consistent results, supporting the robustness of our findings. At this respect, some studies suggest that women with FMS are more likely to experience greater fatigue, a higher number of symptoms, and a greater prevalence of irritable bowel syndrome, whereas men tend to delay seeking medical care and often present with more pronounced physical symptoms [77–79]. Nonetheless, clinical data on male patients remain very limited [78, 79].

In conclusion, personality traits were found to significantly influence the use of mood-stabilizing or regulating medications, but not analgesics, in patients with FMS. Specifically, low levels of neuroticism and/or higher levels of extraversion were associated with a reduced likelihood of antidepressant and anxiolytic use. Additionally, the presence of depressive disorders and low trait extraversion increased the likelihood of opioid use. The severity of anxiety and depression symptoms mediated the relationships between neuroticism and extraversion and the use of anxiolytics and antidepressants. Moreover, the presence of comorbid depressive disorders moderated the association between neuroticism and antidepressant use, such that this association was evident only among patients without a current depression.

Our findings contribute to identifying individual differences in psychological dispositions associated with medication use and symptom severity in patients with FMS. These insights may inform the development of targeted interventions, particularly those incorporating psychotherapeutic and behavioral modification techniques tailored to specific personality traits. As discussed in more detail elsewhere [21], such approaches can enhance adaptive coping strategies by leveraging psychological resources linked to personality profiles. A multidisciplinary treatment model, integrating both psychological and medical approaches, is recommended, as several studies have demonstrated that such interventions are more effective than isolated treatments in managing FMS [80–82]. In light of our findings, behavioral interventions aimed at individuals with high levels of neuroticism may help reduce both symptom severity and reliance on medication. This is particularly important given the established association between neuroticism and increased risk of substance abuse [83], as well as the addictive potential of certain medications commonly used for pain relief. Tailoring interventions to address maladaptive personality traits in FMS patients could reduce emotional burden that exacerbates pain and drives medication dependence, ultimately improving patient outcomes and quality of life. In particular, fostering coping strategies associated with extraversion such as approach-oriented behavior, sociability, optimistic appraisals, positive emotional expression, and assertiveness [84] may help mitigate negative affect and reduce reliance on mood-regulating medications in this population.

## Figures and Tables

**Table 1:** Sociodemographic and clinical variables of patients with fibromyalgia (FMS) and nonclinical control participants (presented as mean ± standard deviation or number of participants and percentage). Group comparison statistics are also included (*F* or *χ*^2^ test).

	**FMS patients (** **n** = 94**)**	**Nonclinical participants (** **n** = 56**)**	**F**/**χ**^2^	**p**	**η**_**p**_^2^
Age	51.71 ± 7.68	50.16 ± 6.81	1.56	0.214	0.010
BMI	28.30 ± 5.29	26.98 ± 4.13	2.60	0.109	0.017
Years of schooling	10.06 ± 4.73	10.27 ± 4.68	0.066	0.798	0.000
State anxiety (STAI)	25.54 ± 14.02	11.82 ± 8.77	43.45	< 0.001	0.227
Trait anxiety (STAI)	44.23 ± 14.96	23.29 ± 11.13	82.51	< 0.001	0.358
Depression (BDI)	28.82 ± 16.01	5.41 ± 5.58	121.18	< 0.001	0.450
Fatigue (FSS)	50.50 ± 12.70	23.67 ± 14.62	138.36	< 0.001	0.485
Insomnia (OSQ)	21.22 ± 11.43	9.43 ± 8.27	45.39	< 0.001	0.235
Sensorial pain (MPQ)	44.36 ± 20.96	4.60 ± 5.90	191.95	< 0.001	0.565
Emotional pain (MPQ)	8.82 ± 5.96	0.80 ± 1.18	98.76	< 0.001	0.400
Evaluative pain (MPQ)	3.70 ± 1.92	1.00 ± 0.85	98.84	< 0.001	0.400
Miscellaneous pain (MPQ)	12.41 ± 7.23	0.79 ± 1.56	140.76	< 0.001	0.487
Comorbid depression	62 (65.95)	1 (1.78)	34.43	< 0.001	—
Comorbid anxiety	55 (58.51)	4 (7.14)	14.28	< 0.001	—
Antidepressants	70 (74.46)	1 (1.78)	74.36	< 0.001	—
Anxiolytics	67 (71.27)	4 (7.14)	57.90	< 0.001	—
Analgesics	67 (71.27)	0 (0)	72.14	< 0.001	—
Opioids	45 (47.87)	0 (0)	38.30	< 0.001	—
Neuroticism	4.50 ± 1.56	1.96 ± 1.63	88.91	< 0.001	0.378
Extraversion	3.50 ± 1.91	4.11 ± 1.79	3.72	0.056	0.025
Psychoticism	2.21 ± 1.13	1.13 ± 1.10	33.42	< 0.001	0.184

Abbreviations: BDI, Beck Depression Inventory; FSS, Fatigue Severity Scale; MPQ, McGill Pain Questionnaire; OSQ, Oviedo Sleep Questionnaire; STAI, State-Trait Anxiety Inventory.

**Table 2:** Correlations between personality traits and clinical variables in patients with fibromyalgia (*p* value in brackets).

	**Neuroticism**	**Extraversion**	**Psychoticism**
Antidepressants	**0.347 (0.001)**	**−0.295 (0.004)**	−0.019 (0.853)
Anxiolytics	**0.250 (0.015)**	**−0.303 (0.003)**	0.036 (0.728)
Analgesics	0.068 (0.513)	−0.093 (0.374)	0.120 (0.251)
Opioids	0.117 (0.262)	**−0.219 (0.034)**	0.027 (0.797)
Anxiety disorders	0.118 (0.255)	**−0.210 (0.042)**	0.063 (0.546)
Depressive disorders	−0.188 (0.069)	−0.189 (0.068)	0.076 (0.468)
State anxiety (STAI)	**0.25 (0.015)**	**−0.23 (0.025)**	−0.05 (0.619)
Trait anxiety (STAI)	**0.46 (< 0.001)**	**−0.34 (0.001)**	−0.06 (0.578)
Depression (BDI)	**0.35 (0.001)**	**−0.33 (0.001)**	0.07 (0.527)
Fatigue (FSS)	**0.21 (0.042)**	−0.10 (0.349)	−0.04 (0.679)
Insomnia (OSQ)	0.13 (0.201)	−0.09 (0.397)	−0.11 (296)
Sensorial pain (MPQ)	0.05 (0.668)	−0.11 (0.291)	**0.44 (< 0.001)**
Emotional pain (MPQ)	0.09 (0.377)	**−0.21 (0.042)**	**0.29 (0.005)**
Evaluative pain (MPQ)	0.11 (0.301)	**−0.24 (0.020)**	**0.22 (0.036)**
Miscellaneous pain (MPQ)	−0.01 (0.931)	−0.05 (0.652)	**0.29 (0.005)**

*Note:* Bold values indicate statistically significant results (*p* < 0.05).

Abbreviations: BDI, Beck Depression Inventory; FSS, Fatigue Severity Scale; MPQ, McGill Pain Questionnaire; OSQ, Oviedo Sleep Questionnaire; STAI, State-Trait Anxiety Inventory.

**Table 3:** Prediction of (a) antidepressant, (b) anxiolytic, and (c) opioid use, and (d) the presence or absence of anxiety disorders in patients with fibromyalgia.

	**Predicted**			
	**Observed**	**Takes**	**No takes**	**Correct (%)**
(a) Antidepressants	No takes	12	12	50.0
	Takes	66	4	94.3
(b) Anxiolytics	No takes	18	9	33.3
	Takes	62	5	92.5
(c) Opioids	No takes	22	27	55.1
	Takes	27	18	60.0
(d) Anxiety disorders	Nonsuffering	11	28	28.2
	Suffering	8	47	85.5

**Table 4:** Results of multiple linear regression analyses identifying personality trait predictors of clinical variables in patients with fibromyalgia.

**Dependent variable**		**Predictor variable**	**β**	**r** ^2^	**t**	**p**
State anxiety (STAI)	1st model	Neuroticism	2.25	0.052	2.47	0.015
Trait anxiety (STAI)	1st model	Neuroticism	4.37	0.198	4.90	< 0.001
	2nd model	Neuroticism	3.80	2.47	4.27	< 0.001
		Extraversion	−1.92		−2.64	0.010
Depression (BDI)	1st model	Neuroticism	3.59	0.112	3.58	0.001
	2nd model	Neuroticism	2.96	0.166	2.95	0.004
		Extraversion	−2.14		−2.62	0.010
Fatigue (FSS)	1st model	Neuroticism	1.71	0.034	2.06	0.042
Sensory pain (MPQ)	1st model	Psychoticism	8.19	0.188	4.75	< 0.001
Emotional pain (MPQ)	1st model	Psychoticism	1.53	0.074	2.91	0.005
	2nd model	Psychoticism	1.58	0.115	3.08	0.003
		Extraversion	−0.70		−2.29	0.024
Evaluative pain (MPQ)	1st model	Extraversion	−0.24	0.047	−2.37	0.020
	2nd model	Extraversion	−0.25	0.090	−2.53	0.013
		Psychoticism	0.39		2.30	0.024
Miscellaneous pain (MPQ)	1st model	Psychoticism	1.84	0.073	2.89	0.005

Abbreviations: BDI, Beck Depression Inventory; FSS, Fatigue Severity Scale; MPQ, McGill Pain Questionnaire; OSQ, Oviedo Sleep Questionnaire; STAI, State-Trait Anxiety Inventory.

**Table 5:** Results of mediation analyses examining the prediction of medication use in patients with fibromyalgia.

**Predictor**	**Mediator**	**Outcome**	**Direct effect**				**Indirect effect**			
			**B**	**SE**	**Z**	**p**	**B**	**Boot SE**	**Boot LLCI**	**Boot ULCI**
Neuroticism	Trait anxiety	Antidepressants	0.318	0.179	1.78	0.075	0.318	0.093	0.072	0.436
Neuroticism	Depression	Antidepressants	0.351	0.173	2.03	0.043	0.193	0.083	0.061	0.386
Extraversion	Trait anxiety	Antidepressants	−0.318	0.162	−1.96	0.050	−0.152	−0.070	−0.321	−0.048
Extraversion	Depression	Antidepressants	−0.307	0.161	−1.91	0.057	−0.158	−073	−0.338	−0.054
Neuroticism	Trait anxiety	Anxiolytics	0.137	0.172	0.798	0.425	0.233	0.099	0.083	0.471
Neuroticism	Depression	Anxiolytics	0.169	0.166	1.02	0.308	0.215	0.090	0.070	0.426
Extraversion	Trait anxiety	Anxiolytics	−0.313	0.154	−2.04	0.042	−0.139	0.067	−0.300	−0.042
Extraversion	Depression	Anxiolytics	−0.298	0.154	−1.93	0.053	−0.158	0.071	−0.332	−0.052

*Note: Z* = standardized test statistic for the direct effect in logistic regression, interpreted as the log odds of the outcome or event occurring (i.e., medication use); Boot = bootstrap estimate derived from 5000 resamples used to compute nonparametric SEs and CIs for indirect (mediation) effects in logistic regression; indirect (mediation) effects were considered unlikely to be due to chance—that is, statistically significant—when the bootstrap confidence intervals did not include zero.

Abbreviations: CI, confidence interval; LLCI, lower limit of the; SE, standard error; ULCI, upper limit of the.

## Data Availability

The data that support the findings of this study are openly available in OSF at https://osf.io/pxt5s/?view_only=35feab3ad71a48f9996ddb37bff5e959.

## References

[1] Wolfe F., Walitt B., Perrot S., Rasker J. J., Häuser W. (2018). Fibromyalgia Diagnosis and Biased Assessment: Sex, Prevalence and Bias. *PLoS One*.

[2] Humphrey L., Arbuckle R., Mease P., Williams D. A., Samsoe B. D., Gilbert C. (2010). Fatigue in Fibromyalgia: A Conceptual Model Informed by Patient Interviews. *BMC Musculoskeletal Disorders*.

[3] Malin K., Littlejohn G. O. (2012). Neuroticism in Young Women With Fibromyalgia Links to Key Clinical Features. *Pain Research and Treatment*.

[4] Wolfe F., Clauw D. J., Fitzcharles M. A. (2010). The American College of Rheumatology Preliminary Diagnostic Criteria for Fibromyalgia and Measurement of Symptom Severity. *Arthritis Care & Research*.

[5] Gálvez-Sánchez C., Duschek S., Reyes del Paso G. A. (2024). Is Reduced Health-Related Quality of Life a Primary Manifestation of Fibromyalgia? A Comparative Study With Rheumatoid Arthritis. *Psychology & Health*.

[6] Martínez M. P., Sánchez A. I., Miró E., Medina A., Lami M. J. (2011). The Relationship Between the Fear-Avoidance Model of Pain and Personality Traits in Fibromyalgia Patients. *Journal of Clinical Psychology in Medical Settings*.

[7] Deus J., Pujol J., Bofill J. (2012). *A Model for Personality*.

[8] Gracely R. H., Petzke F., Wolf J. M., Clauw D. J. (2002). Functional Magnetic Resonance Imaging Evidence of Augmented Pain Processing in Fibromyalgia. *Arthritis & Rheumatism*.

[9] Loggia M. L., Berna C., Kim J. (2014). Disrupted Brain Circuitry for Pain-Related Reward/Punishment in Fibromyalgia. *Arthritis & Rheumatology*.

[10] Montoro C. I., Duschek S., Ladrón M., de Guevara C., Reyes del Paso G. A. (2016). Patterns of Cerebral Blood Flow Modulation During Painful Stimulation in Fibromyalgia: A Transcranial Doppler Sonography Study. *Pain Medicine*.

[11] Bucourt E., Martaillé V., Mulleman D. (2017). Comparison of the Big Five Personality Traits in Fibromyalgia and Other Rheumatic Diseases. *Joint Bone Spine*.

[12] Colangelo N., Bertinotti L., Nacci F. (2004). Dimensions of Psychological Dysfunction in Patients With Fibromyalgia: Development of an Italian Questionnaire. *Clinical Rheumatology*.

[13] López-Espino M., Mingote Adán J. C. (2008). Fibromialgia. *Clínica y Salud*.

[14] Montoro C. I., Reyes del Paso G. A. (2015). Personality and Fibromyalgia: Relationships With Clinical, Emotional, and Functional Variables. *Personality and Individual Differences*.

[15] Montoro C. I., Reyes del Paso G. A., Duschek S. (2016). Alexithymia in Fibromyalgia Syndrome. *Personality and Individual Differences*.

[16] Seto A., Han X., Price L. L., Harvey W. F., Bannuru R. R., Wang C. (2019). The Role of Personality in Patients With Fibromyalgia. *Clinical Rheumatology*.

[17] Coghill R. C. (2010). Individual Differences in the Subjective Experience of Pain: New Insights Into Mechanisms and Models. *Headache*.

[18] Ashgari A., Nicholas M. K. (2006). Personality and Pain-Related Beliefs/Coping Strategies: A Prospective Study. *Clinical Journal of Pain*.

[19] Raphael K. G., Janal M. N., Nayak S., Schwartz J. E., Gallagher R. M. (2006). Psychiatric Comorbidities in a Community Sample of Women With Fibromyalgia. *Pain*.

[20] Keefe F. J., Salley A. N., Lefebvre J. C. (1992). Coping With Pain: Conceptual Concerns and Future Directions. *Pain*.

[21] Davydov D. M., Galvez-Sánchez C., Montoro C. I., Ladrón M., de Guevara C., Reyes del Paso G. A. (2021). Personalized Behavior Management as a Replacement for Medications for Pain Control and Mood Regulation. *Scientific Reports*.

[22] Eysenk H., Eysenk M. (1985). *Personality and Individual Differences: A Natural Science Approach/H*.

[23] Conversano C., Marchi L., Ciacchini R. (2018). Personality Traits in Fibromyalgia (FM): Does FM Personality Exists? A Systematic Review. *Clinical Practice and Epidemiology in Mental Health: CP & EMH*.

[24] Novo R., Gonzalez B., Peres R., Aguiar P. (2017). A Meta-Analysis of Studies With the Minnesota Multiphasic Personality Inventory in Fibromyalgia Patients. *Personality and Individual Differences*.

[25] Silva M. P. S., Carvalho J. F., Rodrigues C. E. M. (2022). Evaluation of Big Five Personality Factors in Women With Fibromyalgia: A Cross-Sectional Study. *Rheumatology International*.

[26] Goubert L., Crombez G., Van Damme S. (2004). The Role of Neuroticism, Pain Catastrophizing and Pain-Related Fear in Vigilance to Pain: A Structural Equations Approach. *Pain*.

[27] Muris P., Meesters C., Van den Hout A., Wessels S., Franken I., Rassin E. (2007). Personality and Temperament Correlates of Pain Catastrophizing in Young Adolescents. *Child Psychiatry and Human Development*.

[28] Malt E. A., Olafsson S., Lund A., Ursin H. (2002). Factors Explaining Variance in Perceived Pain in Women With Fibromyalgia. *BMC Musculoskeletal Disorders*.

[29] Banozic A., Miljkovic A., Bras M. (2018). Neuroticism and Pain Catastrophizing Aggravate Response to Pain in Healthy Adults: An Experimental Study. *Korean Journal of Pain*.

[30] Raselli C., Broderick J. E. (2007). The Association of Depression and Neuroticism With Pain Reports: A Comparison of Momentary and Recalled Pain Assessment. *Journal of Psychosomatic Research*.

[31] Eysenck H. J., Eysenck S. B. (2013). The Biological Basis of Personality. *Personality Structure and Measurement (Psychology Revivals)*.

[32] Park M. S., Lee K. H., Sohn S., Eom J. S., Sohn J. H. (2014). Degree of Extraversion and Physiological Responses to Physical Pain and Sadness. *Scandinavian Journal of Psychology*.

[33] Ferracuti S., De Carolis A. (2005). Relationships Among Eysenck's Extraversion, Rorschach's Erlebnistypus, and Tolerance of Experimental Tonic Pain (Cold Water Pressor Test). *Perceptual and Motor Skills*.

[34] Federman D., Maltz Schwartz R., Amital H. (2019). Extraversion in Women With Fibromyalgia as a Predictor of Better Prognosis: An Intervention Model in Dance Movement Therapy. *Body, Movement and Dance in Psychotherapy*.

[35] Banic B., Petersen-Felix S., Andersen O. K. (2004). Evidence for Spinal Cord Hypersensitivity in Chronic Pain After Whiplash Injury and in Fibromyalgia. *Pain*.

[36] Garaigordobil M., Govillard L. (2016). Síntomas Psicopatológicos en Personas con Fibromialgia: Una Reflexión. *Interdisciplinaria*.

[37] Álvarez M. M. (2016). *Rasgos Psicológicos y Percepción del Dolor en Pacientes con Fibromialgia*.

[38] Cvijetic S., Bobic J., Grazio S., Uremovic M., Nemcic T., Krapac L. (2014). Quality of Life, Personality and Use of Pain Medication in Patients With Chronic Back Pain. *Applied Research in Quality of Life*.

[39] Gibert J. (2006). Antidepresivos, dolor y cáncer. *Psicooncología*.

[40] Rivera J., Vallejo M. A. (2016). Fibromyalgia Is Associated to Receiving Chronic Medications Beyond Appropriateness: A Cross-Sectional Study. *Rheumatology International*.

[41] Kornør H., Nordvik H. (2007). Five-Factor Model Personality Traits in Opioid Dependence. *BMC Psychiatry*.

[42] Kotov R., Gamez W., Schmidt F., Watson D. (2010). Linking “Big” Personality Traits to Anxiety, Depressive, and Substance Use Disorders: A Meta-Analysis. *Psychological Bulletin*.

[43] Sutin A. R., Evans M. K., Zonderman A. B. (2013). Personality Traits and Illicit Substances: The Moderating Role of Poverty. *Drug and Alcohol Dependence*.

[44] Terracciano A., Löckenhoff C. E., Crum R. M., Bienvenu O. J., Costa P. T. (2008). Five-Factor Model Personality Profiles of Drug Users. *BMC Psychiatry*.

[45] Ystrom E., Vollrath M. E., Nordeng H. (2012). Effects of Personality on Use of Medications, Alcohol, and Cigarettes During Pregnancy. *European Journal of Clinical Pharmacology*.

[46] Sutin A. R., Stephan Y., Luchetti M., Terracciano A. (2019). The Prospective Association Between Personality Traits and Persistent Pain and Opioid Medication Use. *Journal of Psychosomatic Research*.

[47] Wolfe F., Smythe H. A., Yunus M. B. (1990). The American College of Rheumatology 1990 Criteria for the Classification of Fibromyalgia. *Arthritis & Rheumatism: Official Journal of the American College of Rheumatology*.

[48] Marques A. P., Santo A. D. S. D. E., Berssaneti A. A., Matsutani L. A., Yuan S. L. K. (2017). Prevalence of Fibromyalgia: Literature Review Update. *Revista Brasileira de Reumatologia*.

[49] First M. B., Spitzer R. L., Gibbon M. (1995). The Structured Clinical Interview for DSM-III-R Personality Disorders (SCID-II). Part II: Multi-Site Test-Retest Reliability Study. *Journal of Personality Disorders*.

[50] Francis L. J., Brown L. B., Philipchalk R. (1992). The Development of an Abbreviated Form of the Revised Eysenck Personality Questionnaire (EPQR-A): Its Use Among Students in England, Canada, the U.S.A. and Australia. *Personality and Individual Differences*.

[51] Sandín B., Valiente R. M., Montes M. O., Chorot P., Germán M. A. S. (2002). Versión Española del Cuestionario EPQR-Abreviado (EPQR-A)(II): Replicación Factorial, Fiabilidad y Validez. *Revista de Psicopatología y Psicología Clínica*.

[52] Lázaro C., Bosch F., Torrubia R., Baños J. E. (1994). The Development of a Spanish Questionnaire for Assessing Pain: Preliminary Data Concerning Reliability and Validity. *European Journal of Psychological Assessment*.

[53] Krupp L. B., LaRocca N. G., Muir-Nash J., Steinberg A. D. (1989). The Fatigue Severity Scale: Application to Patients With Multiple Sclerosis and Systemic Lupus Erythematosus. *JAMA Neurology*.

[54] García J. B., G-Portilla M. P., Martínez P. A., Fernández M. T., Alvarez C. I., Domínguez J. M. (2000). Propiedades Psicométricas del Cuestionario Oviedo de Sueño. *Psicothema*.

[55] Spielberger C. D., Gonzalez-Reigosa F., Martinez-Urrutia A., Natalicio L. F., Natalicio D. S. (1971). The State-Trait Anxiety Inventory. *Revista Interamericana de Psicologia/Interamerican Journal of Psychology*.

[56] Sanz J., Perdigón A. L., Vázquez C. (2003). Adaptación Española del Inventario Para la Depresión de-II (BDI-II): 2. Propiedades Psicométricas en Población General. *Clínica y Salud*.

[57] Hayes A. F. (2013). *Introduction to Mediation, Moderation, and Conditional Process Analysis*.

[58] Bartkowska W., Samborski W., Mojs E. (2018). Cognitive Functions, Emotions and Personality in Woman With Fibromyalgia. *Anthropologischer Anzeiger*.

[59] Rotermann M., Sanmartin C., Hennessy D., Arthur M. (2014). Prescription Medication Use by Canadians Aged 6 to 79. *Health Reports*.

[60] Sullivan M. D. (2018). Depression Effects on Long-Term Prescription Opioid Use, Abuse, and Addiction. *Clinical Journal of Pain*.

[61] Purnamaningsih E. H. (2017). Personality and Emotion Regulation Strategies. *International Journal of Psychological Research*.

[62] Jylhä P., Isometsä E. (2006). The Relationship of Neuroticism and Extraversion to Symptoms of Anxiety and Depression in the General Population. *Depression and Anxiety*.

[63] Jorm A. F., Christensen H., Henderson A. S., Jacomb P. A., Korten A. E., Rodgers B. (2000). Predicting Anxiety and Depression From Personality: Is There a Synergistic Effect of Neuroticism and Extraversion?. *Journal of Abnormal Psychology*.

[64] Watson D., Pennebaker J. W. (1989). Health Complaints, Stress and Distress: Exploring the Central Role of Negative Affectivity. *Psychological Review*.

[65] Paulus D. J., Vanwoerden S., Norton P. J., Sharp C. (2016). From Neuroticism to Anxiety: Examining Unique Contributions of Three Transdiagnostic Vulnerability Factors. *Personality and Individual Differences*.

[66] Yoon K. L., Maltby J., Joormann J. (2013). A Pathway From Neuroticism to Depression: Examining the Role of Emotion Regulation. *Anxiety, Stress & Coping*.

[67] Barnhofer T., Chittka T. (2010). Cognitive Reactivity Mediates the Relationship Between Neuroticism and Depression. *Behaviour Research and Therapy*.

[68] Gaviria A. M., Vinaccia S., Quiceno J. M. (2006). Rasgos de Personalidad, Estrategias de Afrontamiento y Dolor en Pacientes con Diagnóstico de Fibromialgia. *Psicología y Salud*.

[69] Torres X., Bailles E., Valdes M. (2013). Personality Does Not Distinguish People With Fibromyalgia but Identifies Subgroups of Patients. *General Hospital Psychiatry*.

[70] Vassend O., Røysamb E., Nielsen C. S., Czajkowski N. O. (2018). Fatigue Symptoms in Relation to Neuroticism, Anxiety-Depression, and Musculoskeletal Pain. A Longitudinal Twin Study. *PloS One*.

[71] Harkins S. W., Price D. D., Braith J. (1989). Effects of Extraversion and Neuroticism on Experimental Pain, Clinical Pain, and Illness Behavior. *Pain*.

[72] Davydov D. M., Perlo S. (2015). Cardiovascular Activity and Chronic Pain Severity. *Physiology & Behavior*.

[73] Martínez-Correa A., del Paso G. A. R., García-León A., González-Jareño M. I. (2006). Relationship Between Dispositional Optimism/Pessimism and Stress Coping Strategies. *Psicothema*.

[74] Ramírez-Maestre C. R., Velasco Y. V. (2003). Evaluación del Funcionamiento Diario en Pacientes con Dolor Crónico. *Psicología Conductual Revista Internacional de Psicología Clínica de la Salud*.

[75] Clark L. A., Watson D. (1991). Tripartite Model of Anxiety and Depression: Psychometric Evidence and Taxonomic Implications. *Journal of Abnormal Psychology*.

[76] Lysenko N. E., Davydov D. M. (2011). Rating of Textual Descriptions of Violence Scenes Subject to Psychoticism and Sex Differences. *Psikhologicheskii Zhurnal*.

[77] Lysenko N. E., Davydov D. M. (2012). Gender Differences in Regulating Emotions in Response to Text With Violent Content. *Human Physiology*.

[78] Yunus M. B. (2001). The Role of Gender in Fibromyalgia Syndrome. *Current Rheumatology Reports*.

[79] Conversano C., Ciacchini R., Orrù G., Bazzichi M. L., Gemignani A., Miniati M. (2021). Gender Differences on Psychological Factors in Fibromyalgia: A Systematic Review on the Male Experience. *Clinical and Experimental Rheumatology*.

[80] Goldenberg D. L. (2008). Multidisciplinary Modalities in the Treatment of Fibromyalgia. *Journal of Clinical Psychiatry*.

[81] Lera S., Gelman S. M., López M. J. (2009). Multidisciplinary Treatment of Fibromyalgia: Does Cognitive Behavior Therapy Increase the Response to Treatment?. *Journal of Psychosomatic Research*.

[82] Sarzi-Puttini P., Atzeni F., Salaffi F., Cazzola M., Benucci M., Mease P. J. (2011). Multidisciplinary Approach to Fibromyalgia: What Is the Teaching?. *Best Practice & Research Clinical Rheumatology*.

[83] Hokm Abadi M. E., Bakhti M., Nazemi M., Sedighi S., Mirzadeh Toroghi E. (2018). The Relationship Between Personality Traits and Drug Type Among Substance Abuse. *Journal of Research & Health*.

[84] Carver C. S., Connor-Smith J. (2010). Personality and Coping. *Annual Review of Psychology*.

